# How a ferromagnet drives an antiferromagnet in exchange biased CoO/Fe(110) bilayers

**DOI:** 10.1038/s41598-018-37110-8

**Published:** 2019-01-29

**Authors:** M. Ślęzak, T. Ślęzak, P. Dróżdż, B. Matlak, K. Matlak, A. Kozioł-Rachwał, M. Zając, J. Korecki

**Affiliations:** 1 AGH University of Science and Technology, Faculty of Physics and Applied Computer Science, Kraków, Poland; 2 National Synchrotron Radiation Centre SOLARIS, Jagiellonian University, Kraków, Poland; 3 Jerzy Haber Institute of Catalysis and Surface Chemistry PAS, Kraków, Poland

## Abstract

Antiferromagnet/ferromagnet (AFM/FM) bilayers that display the exchange bias (EB) effect have been subjected to intensive material research, being the key elements of novel spintronics systems. In a commonly accepted picture, the antiferromagnet, considered as a rigid material due to its high anisotropy and magnetic hardness, controls the magnetic properties of the ferromagnet, such as a shift of the hysteresis loop or coercivity. We show that this AFM-FM master-slave hierarchy is not generally valid and that the influence of the ferromagnet on the magnetic anisotropy (MA) of the neighbouring antiferromagnet must be considered. Our computer simulation and experimental studies of EB in an epitaxial CoO/Fe(110) bilayer show that the ferromagnetic layer with strong uniaxial magnetic anisotropy determines the interfacial spin orientations of the neighbouring AFM layer and rotates its easy axis. This effect has a strong feedback on the EB effect experienced by the FM layer. Our results show new physics behind the EB effect, providing a route for grafting a desired anisotropy onto the AFM and for precise tailoring of EB in AFM/FM systems.

## Introduction

The ability to modify and detect the spin structure of AFM materials is difficult but interesting from both a fundamental and an application point of view. Two examples among the hottest topics in contemporary spintronics are AFM THz oscillators^1^ and spin orbit torque (SOT) devices^2^. For the realization of an AFM THz oscillator, an AFM with a well-defined easy axis is strongly desired^1^, whereas in the case of SOT devices, the main technical difficulty, namely, the need for an external magnetic field, can be overcome in AFM/FM bilayers^2^ due to the EB^3–5^ effect. From this point of view, the possibility of simultaneously controlling the AFM easy axis and tuning the magnitude of the EB effect in AFM/FM systems is very important. The reorientation of antiferromagnetic moments via an indirect effect of an applied magnetic field was reported^6,7^, where the FM layer followed the direction of the external magnetic field and triggered the formation of the planar domain wall in an exchange-coupled AFM. The importance of this kind of approach, in which the external magnetic field and FM govern the AFM spins, was further beautifully highlighted; see refs^8–12^. Unfortunately, the main drawback of AFM magnetic moments rotating with the external magnetic field is partial or even complete suppression of EB, which is demanded to be large and spontaneous^13^. Another promising approach is to drive the orientation of AFM spins and tune the EB magnitude with epitaxial strain^14^; however, this requires the use of specific substrates or buffer layers, which in turn limit the applications in real devices.

In our report, we consider another possibility to control the interfacial AFM spins by using the intrinsic magnetic properties of the FM layer. We prove that the exchange interaction-driven influence of the ferromagnetic layer on the antiferromagnetic spin structure can be more complex than that suggested by the literature data^6,15^ and relies on a modification of AFM magnetic anisotropy. We show that the ferromagnetic layer with strong and well-defined uniaxial MA can be used to control the magnetic anisotropy and orientation of adjacent AFM magnetic moments and consequently tailor the EB magnitude.

The objects of our research were epitaxial CoO/Fe(110) bilayers grown on W(110). In the Fe/W(110) system, the smooth evolution of the intrinsic uniaxial magnetic anisotropy with the thickness of the Fe layer results in the well-known spin-reorientation-transition (SRT)^16–20^, in which changes in the MA strength lead to the switching of the Fe easy axis. Specifically, the Fe magnetization switches from the [1−10] to the [001] in-plane direction when the Fe thickness increases above a critical value that can be tuned in the range of 100 ± 50 Å. Such an SRT, albeit modulated by the EB effect, is also observed in CoO/Fe(110) bilayers. To trace the evolution of the interfacial AFM easy axis and the EB effect strength with the change of the effective in-plane MA of the FM layer, the magnetic hysteresis loops were measured and carefully analysed as a function of the Fe thickness.

## Results

**Figure 1:**
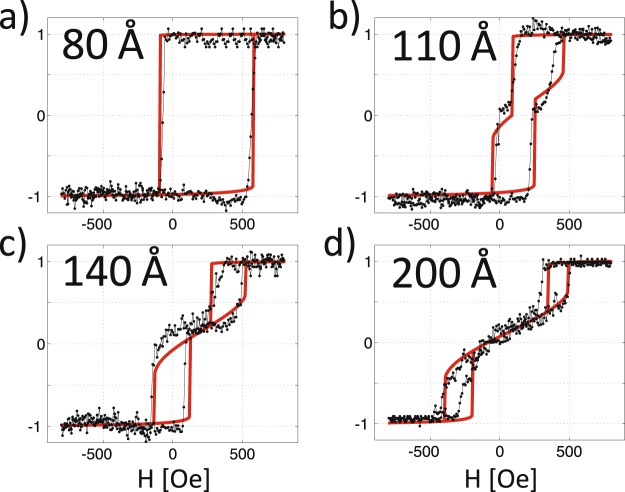
Normalized magnetic hysteresis loops measured using the MOKE (at T = 183 K) and simulated for selected Fe thicknesses: (**a**) 80 Å, (**b**) 110 Å, (**c**) 140 Å and (**d**) 200 Å. The external magnetic field was applied along the [1−10] direction in the Fe(110) plane.

**Figure 2:**
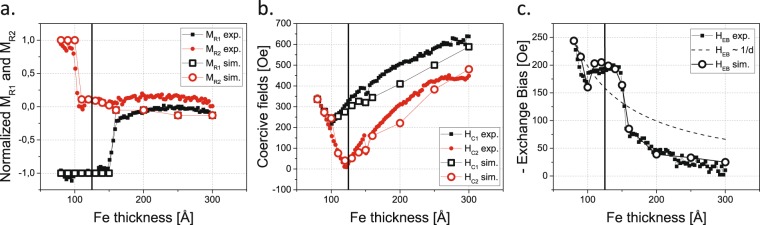
Thickness dependence of magnetic parameters derived from MOKE measurements (full symbols) and simulations within a coherent rotation model of magnetization reversal (open symbols) for an external magnetic field along the [1–10] direction in the Fe(110) plane, at 183 K. (**a**) The normalized remanent magnetizations M_R1_ and M_R2_ corresponding to the states after saturation in positive and negative external magnetic fields, respectively. (**b**) Absolute values of coercive fields H_c1_ and H_c2_. (**c**) Shift field H_EB_ compared with 1/d_Fe_ (dashed line) dependence. The solid vertical lines in a–c mark the critical thickness of SRT in Fe.

In Fig. 1, selected hysteresis loops acquired with a magneto-optic Kerr effect (MOKE) microscope at T = 183 K after cooling the sample in a remanent state are shown for *d*_*Fe*_ = 80, 110, 140 and 200 Å. The positive direction of the external magnetic field in Fig. 1 corresponds to its antiparallel orientation with respect to the remanent Fe magnetization direction during the cooling process. From these hysteresis loops, full *d*_*Fe*_ dependences of the normalized remanent magnetization M_R1_ and M_R2_ (Fig. 2a, filled symbols), coercive fields H_c1_ and H_c2_ (Fig. 2b, filled symbols) and shift field H_EB_ (Fig. 2c, filled symbols), as defined in ref.^15^, were determined.

For d_Fe_ below ~100 Å, EB-shifted square hysteresis loops, characteristic of the easy magnetization direction, are observed (Fig. 1a). Therefore, the values of M_R1_ and M_R2_ are almost thickness independent and equal to −1 and 1, respectively (Fig. 2a). At d_Fe_ = 100 Å, the hysteresis loops begin to evolve towards hard magnetization loops (see Fig. 1b,c), which means that the CoO/Fe bilayer enters the SRT region. These split loops are also EB shifted, and for this reason, initially, only one of the remanence states exhibits a remanence M_R2_ close to zero, whereas the second state is characterized by M_R1_ = −1, as shown in Fig. 2a. This situation persists up to d_Fe_ ~ 150 Å. At this Fe thickness, the shift of the hysteresis loops is already markedly reduced, and MA is enhanced enough to result in M_R1_ ≈ M_R2_ ≈ 0, as observed for typical hard axis loops in Fe on W(110)^21^, similar to the one presented in Fig. 1d. In addition an analysis of the thickness driven evolution of the hysteresis loop shape allows to determine the critical thickness of SRT in the CoO/Fe system, which we found to be d_c_ = 125 Å. In particular, at an external magnetic field value compensating the EB field, H = H_EB_, the normalized Kerr rotation switches its value from 1 to 0 at the critical Fe thickness.

We now focus on the H_EB_(d_Fe_) dependence presented in Fig. 2c (squares) alongside the 1/d_Fe_ curve (dashed line), which typically describes the interfacial EB effect^22^. Two uncommon features in the H_EB_ dependence on d_Fe_ are to be noted. The first is the significant enhancement of the H_EB_ value for d_Fe_ in the range 100–150 Å, with a drastic drop occurring at a d_Fe_ of approximately 150 Å. The second uncommon feature is that even outside the 100–150 Å d_Fe_ range, the experimental dependence definitely does not follow 1/d_Fe_. It has to be noted that the critical SRT thickness d_c_ (marked by the solid vertical lines in Fig. 2a–c) is located roughly in the middle of the region defined by the critical points where M_R1_ and M_R2_ become zero. Similarly in Fig. 2c, d_c_ is situated in the center of the plateau of the H_EB_(d_Fe_) dependence. This unusual H_EB_ vs d_Fe_ dependence has to be discussed in view of available theoretical models and similar literature data on EB. The 1/d^1.9^ dependence of H_EB_ for thick FM films was theoretically predicted^23^ and 1/d^2^ dependence was experimentally observed^24^. Such thickness dependence of EB involved the formation of planar domain walls in both AFM and FM layers. Invalidity of such modelling of our experimental results can be seen without any numerical analysis at the low thickness limit, where the strong deviation of H_EB_ vs d_Fe_ dependence from the ~1/d_Fe_ proportionality is determined from the perfectly square hysteresis loops, excluding the existence of any non-collinear Fe spin structures.

To identify the physical origin of this unusual EB behaviour, we performed simulations of the magnetic hysteresis loops for the entire studied range of d_Fe_. The magnetization reversal was described within the coherent rotation model; see ‘Supplemental Material 1’ for details. For each d_Fe_, the values of Fe magnetic anisotropy constants and Φ_CoO_, the angle between the projection of the CoO spins on the Fe(110) plane and the Fe[1−10] direction, were tuned to obtain the best fit between the simulated and experimental hysteresis loops. The good agreement between the black dotted (measured) and red solid (calculated) hysteresis loops in Fig. 1 is to be noted.

**Figure 3:**
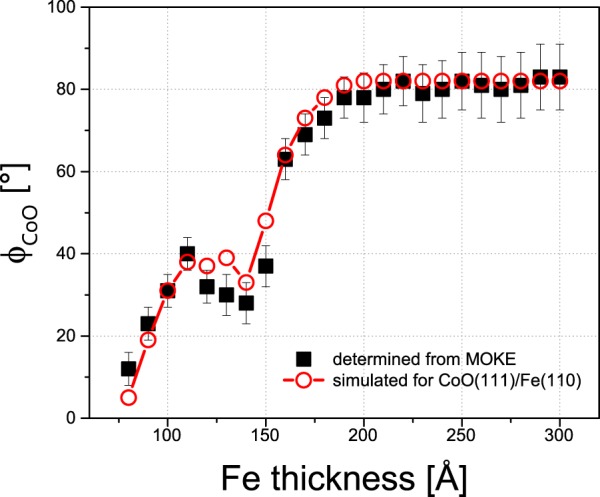
Thickness dependence of Φ_CoO_ with respect to the in-plane Fe[1−10] direction as obtained from MOKE data analysis (black squares). For comparison, the results of the equilibrium remanent state simulations, performed for the CoO(111)/Fe(110) system (red open points), are shown.

The results from such loop modelling, which show perfect qualitative and good quantitative agreement with the MOKE data, are presented in Fig. 2 (large open symbols). The characteristic dependencies of M_R1_, M_R2_, H_C1_, and H_C2_ on d_Fe_ that were observed with the MOKE are reproduced by the simulations (Fig. 2a,b). More importantly, the unusual thickness dependence of H_EB_ is also perfectly reproduced using the coherent rotation model of magnetization reversal parameterized by Fe MA constants and Φ_CoO_. The obtained dependence of Φ_CoO_ on the Fe thickness is plotted in Fig. 3 with black squares.

With increasing d_Fe_, the effective MA of the Fe layer evolves, and its easy axis reorients from the [1−10] to the bulk-like [001] direction. Simultaneously, with this SRT in the ferromagnet, another spin reorientation occurs at the AFM/FM interface. Namely, the CoO spins rotate away from the [1−10] towards the [001] in-plane direction, as presented in Fig. 3. In the middle of this “double-SRT (d_c_ = 125 A),” the effective MA of the ferromagnet becomes zero, and therefore, the driving force for the SRT in CoO vanishes. This in turn leads to a deviation from the hypothetical monotonous evolution of Φ_CoO_ from [1−10] to [001], which is manifested by the deep change in the dependence of Φ_CoO_ on d_Fe_ around 100–150 Å. The SRT at the interface of the AFM CoO is therefore induced by the in-plane MA evolution of the FM Fe layer and is reflected in the anomalous dependence of H_EB_ on d_Fe_ presented in Fig. 2c. We conclude that Fe magnetization acts as a local external magnetic field, which imprints the magnetic anisotropy in the adjacent CoO layer. Hence, both the interfacial orientation of AFM magnetic moments and the spontaneous EB effect strength can be tuned by the thickness-driven SRT process in the Fe film. Contrary to previous reports, no external magnetic field is necessary. Since additional magnetic anisotropy is “grafted” into the AFM, its magnetic moments do not rotate with the applied magnetic field, and thus, the EB effect is not reduced. Consequently, the Φ_CoO_ angle remains unchanged for each given hysteresis curve (as the induced AFM anisotropy), whereas its thickness dependence is strongly affected by the SRT process in the Fe layer.

**Figure 4:**
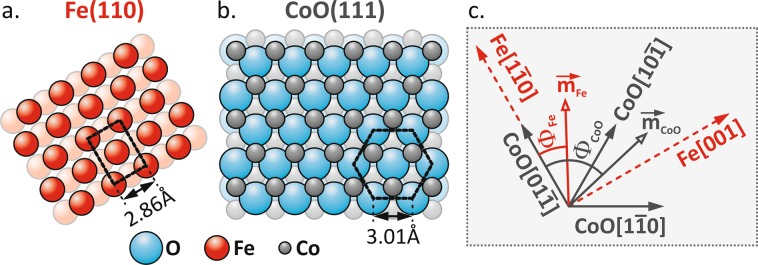
Ball models of (**a**) Fe(110) and (**b**) CoO(111) surfaces. The relative Fe and CoO in-plane directions as concluded from LEED analysis are sketched in (**c**), along with the definitions of the Φ_Fe_ and Φ_CoO_ angles.

To further support the above interpretation and to shed light on the mechanism responsible for the magnetic anisotropy imprinted by the Fe layer in the CoO film, we performed simulations of the d_Fe_ thickness-driven evolution of the remanent equilibrium state in the CoO(111)/Fe(110) system. These simulations are based on the global minimization of the free energy E(d_Fe_, Φ_Fe_, Φ_CoO_); see ‘Supplemental Material 1’ for details. We consider three AFM MA contributions that correspond to the three 〈011〉 crystallographic directions in the threefold coordinated CoO(111) plane (Fig. 4); see ‘Supplemental Material 2’ for the corresponding low energy electron diffraction (LEED) analysis. We assume that the strength of these MA contributions, defined by MA constants K_CoO[01–1]_, K_CoO[10–1]_ and K_CoO[1–10]_, is proportional to the strength and depends on the sign of the effective in-plane MA of the Fe layer. Specifically, when the Fe layer is in the state before the SRT and thus its effective MA prefers the Fe[1–10] easy axis of magnetization, all three AFM contributions, K_CoO[01–1]_, K_CoO[10–1]_ and K_CoO[1–10]_, are triggered because all 〈011〉 directions in the CoO(111) plane have non-zero projections along the Fe[1–10] direction. Since the CoO[01–1] and Fe[1–10] directions are parallel, we assume the strongest contribution of the K_CoO[01–1]_ constant for thin Fe films (Fig. 3 in ‘Supplemental Material 1’). After the SRT in Fe occurs, Fe[001] becomes the easy axis of the magnetization. Consequently, for thicker Fe films, we assume that both the CoO[10–1] and CoO[1–10] directions are equally activated because Fe[001] lies exactly between these directions. At the same time, the CoO[01–1] contribution is diminished, as it is perpendicular to the Fe[001] direction.

A detailed description of the input parameters used in the described simulations can be found in ‘Supplemental Material 1.’ Here, we note that their results, shown with red open points in Fig. 3, almost perfectly mimic the Φ_CoO_(d_Fe_) dependence obtained from the MOKE analysis. The presented simulations lead to the scenario in which the Fe layer, depending on its own magnetic anisotropy, locally ‘activates’ the directionally nearest CoO magnetic anisotropy axes when passing the Neel temperature of CoO. Our simulations indicate that the magnetic anisotropy of CoO induced by the proximity of the Fe layer is clearly not uniaxial but must be composed of more than one contribution. Such a state can be realized via the formation of antiferromagnetic domains in CoO, and their areal contributions define the magnitude of the corresponding anisotropy constant. In this way, the smooth evolution of FM MA triggers the continuous and non-monotonous rotation of interfacial AFM magnetic moments.

**Figure 5:**
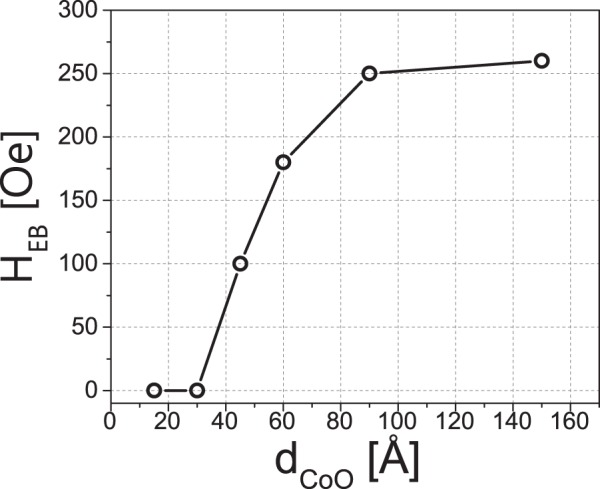
H_EB_ vs d_CoO_ dependence as determined from MOKE data for the d_Fe_ = 80 Å, at 183 K.

Finally, the X-ray Magnetic Linear Dichroism (XMLD) technique was applied to directly observe CoO spin reorientation transition that follows the SRT in Fe layer. For that purpose an epitaxial Fe(110) films with two distinct thicknesses corresponding to the [1–10] (d_Fe_ = 50 Å) and [001] (d_Fe_ = 200 Å) Fe spin orientation, coated by the CoO film, was studied for the d_CoO_ = 90 Å and 45 Å. The choice of CoO thicknesses was made basing on the H_EB_ vs d_CoO_ dependence studied with MOKE for the d_Fe_ = 80 Å at 183 K, see Fig. 5.

It is clear that exchange bias develops above d_CoO_ = 30 Å and saturates at ~90 Å. In order to observe the XMLD effect, the X-ray Absorption Spectroscopy (XAS) spectra were *ex-situ* acquired at the XAS end station of National Synchrotron Radiation Centre Solaris in Kraków, in the energy range that covers the L3 absorption edge of Co. It has to be noted the subsequent *ex-situ* MOKE measurements indicated no change of exchange bias and magnetic anisotropy of Fe/CoO system. The direction of the incoming X rays was parallel to the sample normal and its linear polarization was parallel to the Fe[1–10] direction (**E** II [1–10]). In such geometry, for the given CoO thickness, a signature of the CoO spin reorientation can be directly observed from the comparison of the XAS spectra corresponding to CoO/50 Å Fe and CoO/200 Å Fe sample regions.

**Figure 6:**
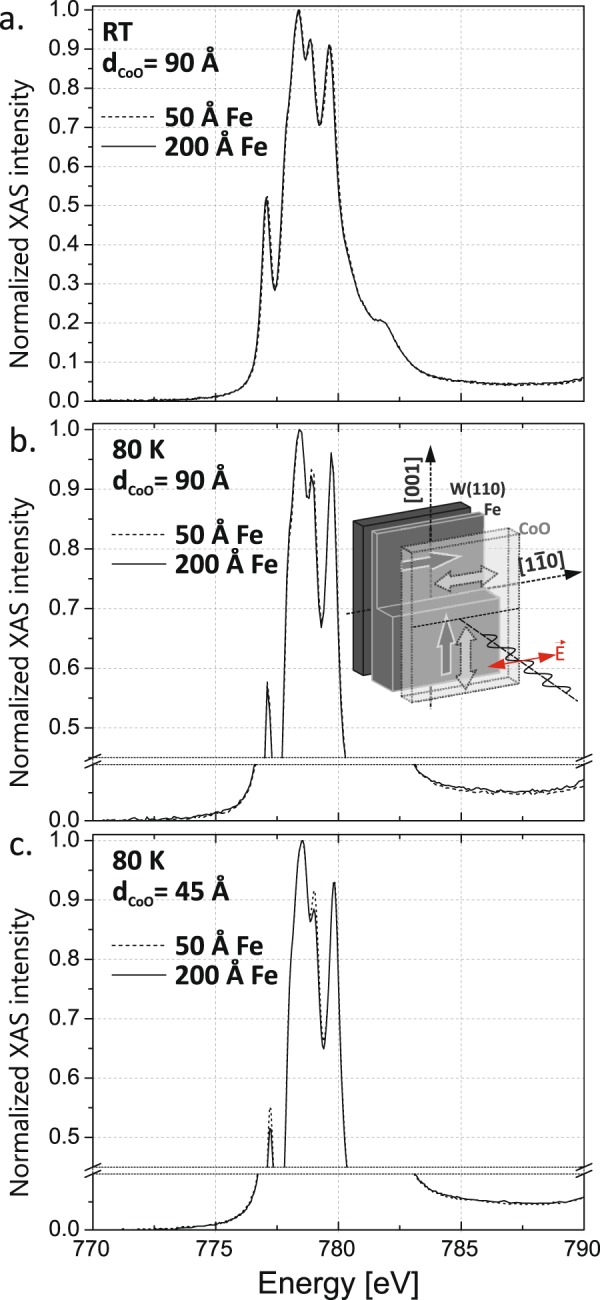
XAS spectra acquired around the L3 absorption edge of Co. Details of the XAS measurements geometry are shown in the inset in b. The direction of the incoming X rays is parallel to the sample normal and its linear polarization is parallel to the Fe[1–10] direction (**E** II [1–10]).

In case of both studied CoO thicknesses, d_CoO_ = 90 Å and 45 Å, the room temperature (RT) XAS spectra were exactly identical, see Fig. 6 a for d_CoO_ = 90 Å, as expected above CoO Neel temperature. At T = 80 K, the difference of XAS spectra for the two Fe thicknesses develops with decreasing thickness of CoO. In particular for d_CoO_ = 90 Å the difference of peak intensities for 779 eV energy is hardly visible, see Fig. 6b. However for the sample with d_CoO_ = 45 Å we observed a significant change of the XAS spectrum for the two Fe thicknesses, as shown in the Fig. 6c. Following analysis of the XMLD measurements presented in^25,26^, for the CoO spins along the [1–10] direction (vector **E** parallel to [1–10]) the XAS peaks around 777.2 eV and 779 eV have higher intensities than in the case of CoO spins along the [001] direction (vector **E** orthogonal to [1–10]), indicating the in-plane reorientation of CoO spins induced by Fe SRT. The comparison of the XAS results for the two CoO thicknesses indicates the interfacial character of the CoO magnetic anisotropy modifications induced by Fe layer. In case of 90 Å thick CoO film the XAS intensity is dominated by about ~50 Å thick topmost CoO surface layer, as confirmed by almost complete damping of the XAS intensity measured at the Fe L3 absorption edge (not shown). It is clear that for such thick CoO layer the interfacial contribution to the XAS spectra is small and therefore only a tiny XMLD effect could be detected. Moreover the XAS measurements performed as a function of incidence angle with respect to the sample normal (not shown) revealed the in-plane orientation of CoO spins in our CoO/Fe system, as the discussed above difference in XAS intensities for both Fe thicknesses is maximal for the normal incidence geometry. The XAS results together with presented MOKE data and simulations are a strong evidence of the CoO in-plane spin reorientation transition induced by the underlying Fe layer.

## Conclusions

In conclusion, we highlight the possibility to simultaneously control the interfacial spin axis of an antiferromagnet and to tailor the EB effect in FM/AFM systems with the well-defined MA of a ferromagnet. Moreover, the reported magnetic anisotropy of the AFM induced by an FM layer well explains the presence of a spontaneous EB in many FM/AFM systems. This kind of MA engineering could be used in future experiments with both self-organized and patterned nanostructures. One such promising example is Fe islands on W(110)^27^. In specially prepared Fe/W samples, [1−10] as well as [001] magnetized nanostructures can exist. In addition, another possibility, i.e., nanostructures with coexisting [1−10] and [001] magnetic domains, has also been reported^19,27^. When such nanostructures are capped with CoO, a strong local sensitivity of the EB effect and CoO spin orientation to the magnetic state of a given Fe island can be expected.

## Methods

### Sample preparation

A wedged Fe(110) film of thickness d_Fe_ ranging from 80 to 300 Å was grown on an atomically clean W(110) single crystal at room temperature using molecular beam epitaxy (MBE) followed by annealing at 675 K. This produced high-quality Fe films with an atomically smooth (110) surface. Next, a 90-Å CoO adlayer was grown on the Fe(110) wedge film by reactive deposition of cobalt in an O_2_ atmosphere (partial pressure of 5 × 10^−7^ mbar) at a temperature of 470 K.

### Structural properties: LEED analysis

The low-energy electron diffraction (LEED) technique was used to study the surface structure of the Fe(110) and CoO/Fe(110) films. The details of the structural characterization are presented in Supplemental Material 2.

### Magnetic properties: MOKE measurements

The magnetic properties of the CoO/Fe(110) system were studied *in situ* as a function of the Fe thickness using longitudinal magneto-optic Kerr effect (MOKE) imaging. The field of view of the MOKE system was tuned to cover the whole sample, which was 8 mm in diameter. A series of MOKE images was taken as a function of the external magnetic field *H*, which was applied along the Fe[1−10] in-plane direction. Magnetic hysteresis loops could be extracted for any selected sample region of interest (ROI), which can be as small as one pixel. This method recently proved to be very efficient in MA studies, and in EB studies, its advantages are even more significant. For the wedged sample, a single cooling procedure followed by the acquisition of a single MOKE “movie” as a function of the external magnetic field provided a full data set for the magnetization-reversal measurements. Moreover, all hysteresis loops were obtained under exactly the same experimental conditions, i.e., at the same sample temperature, with the same possible sample misalignments and with the same magneto-optical artefacts, if any. The size of a single ROI along the Fe-wedge gradient was 50 µm, which corresponded to an averaging of the loops over a finite Fe-thickness difference of approximately 2 Å.

### Magnetic properties: XMLD measurements

The X-ray Absorption Spectroscopy (XAS) spectra were *ex-situ* measured at the XAS end station of National Synchrotron Radiation Centre Solaris in Kraków, in the energy range that covers the L3 absorption edge of Co. The direction of the incoming X rays was parallel to the sample normal and its linear polarization was parallel to the Fe[1–10] direction (**E** II [1–10]). In such geometry, for the given CoO thickness, a signature of the CoO spin reorientation can be directly observed from the comparison of the XAS spectra corresponding to CoO/50 Å Fe and CoO/200 Å Fe sample regions.

### Simulations: global and local energy minimum

Two kinds of magnetic simulations were performed. The simulated magnetic hysteresis loops were obtained from the minimization of the free enthalpy density, G(Φ_Fe_, Φ_CoO_ = const.), for each external magnetic field H value. The simulation of the Fe thickness-driven evolution of the remanent equilibrium state in the CoO(111)/Fe(110) system was based on the global minimization of the free energy E(Φ_Fe_, Φ_CoO_) for each given d_Fe_. For details of the simulations, please see ‘Supplemental Material 1’.

This work is part of the scientific activities of the CERIC-ERIC internal project MAG-ALCHEMI. This work was supported by the National Science Center Poland (NCN) under Project No. 2011/02/A/ST3/00150 and (partially) by the AGH UST statutory tasks No. 11.11.220.01/6 within a subsidy of the Ministry of Science and Higher Education. A.K-R. was supported by the “Antiferromagnetic proximity effect and development of epitaxial bimetallic antiferromagnets –two routes towards next-generation spintronics” project which is carried out within the Homing programme of the Foundation for Polish Science co-financed by the European Union under the European Regional Development Fund.

## Supplementary information

Supplemental material 1

Supplemental material 2
