# Supplementary material for: How a ferromagnet drives an antiferromagnet in exchange biased CoO/Fe(110) bilayers
Source: Sci Rep. 2019 Jan 29;9:889. doi: 10.1038/s41598-018-37110-8 (PMC6351541; doi:10.1038/s41598-018-37110-8)
Supplement: Supplementary file 1 — Supplemental material 1 [file 41598_2018_37110_MOESM1_ESM.pdf]

How a ferromagnet drives an antiferromagnet in exchange biased in CoO/Fe(110) bilayers

M. Ślęzak[1*], T. Ślęzak[1], P. Dróżdż[1], B. Matlak[1], K. Matlak[1], A. Kozioł-Rachwał[1], M. Zając[2], J. Korecki[1,3]

[1] AGH University of Science and Technology, Faculty of Physics and Applied Computer Science, Kraków, Poland

[2] National Synchrotron Radiation Centre SOLARIS, Jagiellonian University, Kraków, Poland

[3] Jerzy Haber Institute of Catalysis and Surface Chemistry PAS, Kraków, Poland

Simulations I: magnetic hysteresis loops from local energy minimum

For each given Fe thickness d_{Fe}, simulated magnetic hysteresis loops are obtained from the minimization of the free enthalpy density, $G(\Phi_{Fe}, \Phi_{CoO} = \text{const.})$, in the domain of the Φ_{Fe} angle defining the orientation of Fe magnetization with respect to the Fe[1−10] in-plane direction as a function of the external magnetic field, H:

$$G(d_{Fe}, \Phi_{Fe}, \Phi_{CoO}) = E_{CoO}(\Phi_{CoO}, d_{Fe}) + E_{CoO\text{-}Fe}(\Phi_{CoO}, \Phi_{Fe}) + E_{Fe}(\Phi_{Fe}, d_{Fe}) + E_H. \qquad (1)$$

The first three terms in equation (1) describe the in-plane magnetic anisotropy (MA) contribution to the free energy of the CoO/Fe bilayer. The exchange energy at the interface, $E_{CoO\text{-}Fe}$, and the Fe MA energy, E_{Fe} are described by the following formulas:

$$E_{CoO\text{-}Fe}(\Phi_{CoO}, \Phi_{Fe}) = - K_{EB}/d_{Fe} \cos(\Phi_{Fe} - \Phi_{CoO}), \qquad (2)$$

$$E_{Fe}(\Phi_{Fe}, d_{Fe}) = A \cos^2(\Phi_{Fe}) + B \cos^4(\Phi_{Fe}), \qquad (3)$$

where K_{EB} is the CoO/Fe exchange-coupling constant. The Φ_{CoO} angle is the angle between the projection of the CoO spins on the Fe(110) plane and the Fe[1−10] direction. For definition of the angles used in the simulations, see Fig. 1 below.

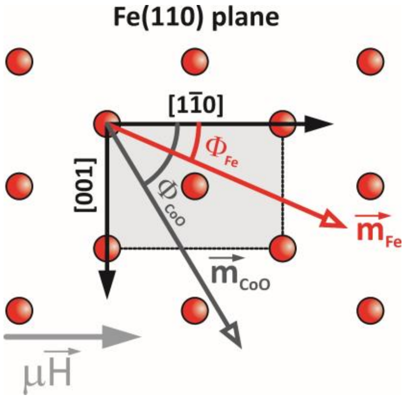

Fig. 1 A schematic sketch of angles used in the simulations.

The second- and fourth-order effective MA constants of Fe, A and B, respectively, can be defined in terms of the volume and surface MA contributions:

$$A = K_{v,p} - K_{s,p}/d_{Fe}, \qquad (4)$$

$$B = K_{v,pp} - K_{s,pp}/d_{Fe}. \qquad (5)$$

In Equations 4 and 5, $K_{v,p}$ and $K_{v,pp}$ are the second- and fourth-order volume constants of the in-plane magnetic anisotropy, while $K_{s,p}$ and $K_{s,pp}$ denote their surface analogues.

For given d_{Fe} and Φ_{CoO} independent of the external field, the CoO MA energy term, E_{CoO}, maintains a constant value for each external magnetic field value H, and therefore, it has no influence on the shape of simulated hysteresis curves. The physical meaning and importance of the E_{CoO} term will be discussed in the next section of this supplemental material.

The last term in Eq.1 is a Zeeman term, which describes the interaction with the external magnetic field H:

$$E_H = - M_s H \cos(\Phi_{Fe}), \qquad (6)$$

where M_s is the Fe saturation magnetization.

For all simulated hysteresis loops, K_{EB} was fixed at 0.5 mJ/m^2, which was found to provide the best fit to the experimental results. For each d_{Fe} (which means for each particular hysteresis curve), the values of A, B and Φ_{CoO} were tuned to obtain the best fit between the simulated and experimental hysteresis loops. In all simulations, a 5° misalignment between the Fe[1−10] direction and the external magnetic field was assumed to account for the observed asymmetric

shape of the hysteresis loops. The dependence of A and B on the inverse Fe thickness ($1/d_{Fe}$) is plotted in Fig. 2.

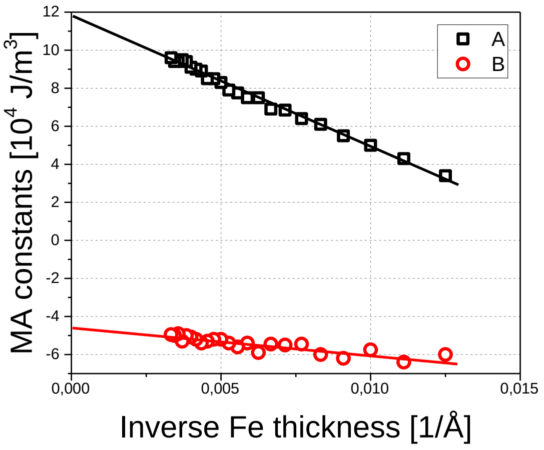

Fig. 2 Inverse-thickness dependence of A and B constants.

The magnetic anisotropy constants A and B show a linear dependence on $1/d_{Fe}$. From linear regression, as shown in Figure 2, it is found that:

$$K_{v,p} = 11.8 \times 10^4 \text{ J/m}^3,$$
$$K_{s,p} = 0.7 \text{ mJ/m}^2,$$
$$K_{v,pp} = -4.6 \times 10^4 \text{ J/m}^3$$
$$K_{s,pp} = 0.1 \text{ mJ/m}^2. \quad (7)$$

Simulations II: the remanent equilibrium state in the CoO(111)/Fe(110) system from the global minimization of the free energy

In the following, we describe the details of the input parameters used in the simulations shown with red open symbols in Fig. 3 of the main text.

The simulation of the Fe thickness-driven evolution of the remanent equilibrium state in the CoO(111)/Fe(110) system is, for each given d_{Fe}, based on the global minimization of the free energy $E(d_{Fe}, \Phi_{Fe}, \Phi_{CoO})$ in the 2-dimensional space of angles Φ_{Fe} and Φ_{CoO}. We define the free energy E using formula 1, where the external magnetic field is fixed and equal to zero, $H = 0$.

We model the CoO MA energy term, E_{CoO}, using the following formula:

$$E_{CoO}(\Phi_{CoO}) = - K_{CoO\,[01\text{-}1]} \cos^2(\Phi_{CoO}) - K_{CoO\,[10\text{-}1]} \cos^2(\Phi_{CoO} - 60°) - K_{CoO\,[1\text{-}10]} \cos^2(\Phi_{CoO} - 120°). \quad (8)$$

The three terms in formula 8, defined by CoO MA constants $K_{CoO[01-1]}$, $K_{CoO[10-1]}$ and $K_{CoO[1-10]}$, correspond to the three <011> crystallographic directions in the threefold coordinated CoO(111) plane; see ‘Supplemental material 2’ for the corresponding LEED analysis. We assume that these MA contributions and thus the values of CoO MA constants $K_{CoO[01-1]}$, $K_{CoO[10-1]}$ and $K_{CoO[1-10]}$ are proportional to the strength and depend on the sign of the effective in-plane MA of the Fe layer. Specifically, when the Fe layer is in the state before the SRT and thus its effective MA prefers the Fe[1-10] easy axis of magnetization, all three AFM contributions, $K_{CoO[01-1]}$, $K_{CoO[10-1]}$ and $K_{CoO[1-10]}$, are non-zero (Fig. 3) because all CoO(111) <011> directions have non-zero projections along the Fe[1-10] direction.

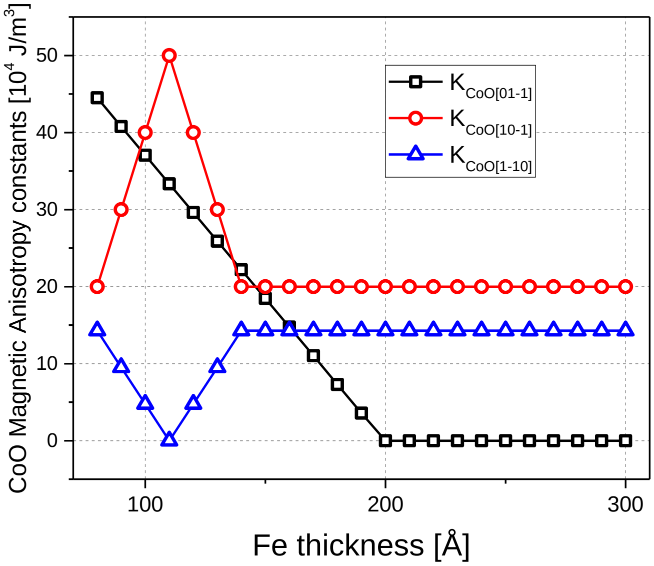

Fig. 3. CoO magnetic anisotropy constants $K_{[01-1]}$, $K_{[10-1]}$ and $K_{[1-10]}$ vs. Fe thickness assumed in the simulations presented in the main text.

Since the CoO[01-1] and Fe[1-10] directions are parallel, we assume the strongest contribution of the $K_{CoO[01-1]}$ constant for thin Fe films (Fig. 3, black squares). With increasing Fe thickness, the strength of the Fe[1-10] MA is continuously decreasing, and consequently, we assume the linear decrease of the $K_{CoO[01-1]}$ constant. Two AFM MA contributions, $K_{CoO[10-1]}$ and $K_{CoO[1-10]}$, are modelled in the following way. When the effective Fe magnetization rotates from Fe[1-10]

towards the Fe[001] direction, it initially becomes directionally (i) closer to CoO[10-1] and (ii) further from the CoO[1-10] direction. To include these two facts in our simulations, we assume (i) an initial increase of the $K_{CoO[10-1]}$ constant (red circles in Fig. 3) and (ii) an initial decrease of the $K_{CoO[1-10]}$ constant (blue triangles in Fig. 3). Around the middle of the SRT in Fe, the effective angles between the Fe magnetization and (i) CoO[10-1] and (ii) CoO[1-10] directions start to (i) decrease and (ii) increase, as do the corresponding AFM MA constants used as input parameters in the simulations. After the SRT in Fe occurs, its MA supports the Fe[001] to be the easy axis of the magnetization, and thus, the $K_{CoO[01-1]}$ constant is reduced to zero. On the other hand, both the CoO[10-1] and CoO[1-10] directions are almost equally 'activated' by the Fe[001] magnetization that now lies between these two directions; therefore, the $K_{CoO[10-1]}$ and $K_{CoO[1-10]}$ constants are reduced back to their initial values.
