# Supplementary material for: How a ferromagnet drives an antiferromagnet in exchange biased CoO/Fe(110) bilayers
Source: Sci Rep. 2019 Jan 29;9:889. doi: 10.1038/s41598-018-37110-8 (PMC6351541; doi:10.1038/s41598-018-37110-8)
Supplement: Supplementary file 2 — Supplemental material 2 [file 41598_2018_37110_MOESM2_ESM.pdf]

How a ferromagnet drives an antiferromagnet in exchange biased in CoO/Fe(110) bilayers

M. Ślęzak[1*], T. Ślęzak[1], P. Dróżdż[1], B. Matlak[1], K. Matlak[1], A. Kozioł-Rachwał[1], M. Zając[2], J. Korecki[1,3]

[1] AGH University of Science and Technology, Faculty of Physics and Applied Computer Science, Kraków, Poland

[2] National Synchrotron Radiation Centre SOLARIS, Jagiellonian University, Kraków, Poland

[3] Jerzy Haber Institute of Catalysis and Surface Chemistry PAS, Kraków, Poland

Structural properties of Fe(110) and CoO/Fe(110) films grown on W(110)

The exemplary LEED pattern of the uncovered Fe(110) surface is shown in Fig. 1a. Sharp diffraction spots indicate a smooth unreconstructed (110) surface. From the LEED patterns acquired across the Fe wedge, we determined the thickness dependence of the in-plane lattice spacing a_{001} along the Fe[001] direction for the Fe(110) surface (Fig. 1b). In the entire studied Fe thickness range, we found a_{001}=2.88±0.02 Å, which corresponds to an almost fully relaxed Fe(110)/W film when compared to 2.86 Å for bulk iron. The LEED pattern from the surface of the CoO-covered Fe(110)/W(110) sample shown in Fig. 1c indicates a hexagonal CoO(111) surface structure throughout the underlying Fe film. Scanning of a CoO surface with the electron beam revealed within the LEED accuracy a homogenous CoO surface structure, with in-plane lattice spacing $a_{1\text{-}10}$ = 3.01±0.03 Å. From the LEED patterns, we also conclude that the Fe[1-10] and CoO[01-1] in-plane directions are parallel, while Fe[001] lies between the CoO[10-1] and CoO[1-10] directions (Fig. 1d).

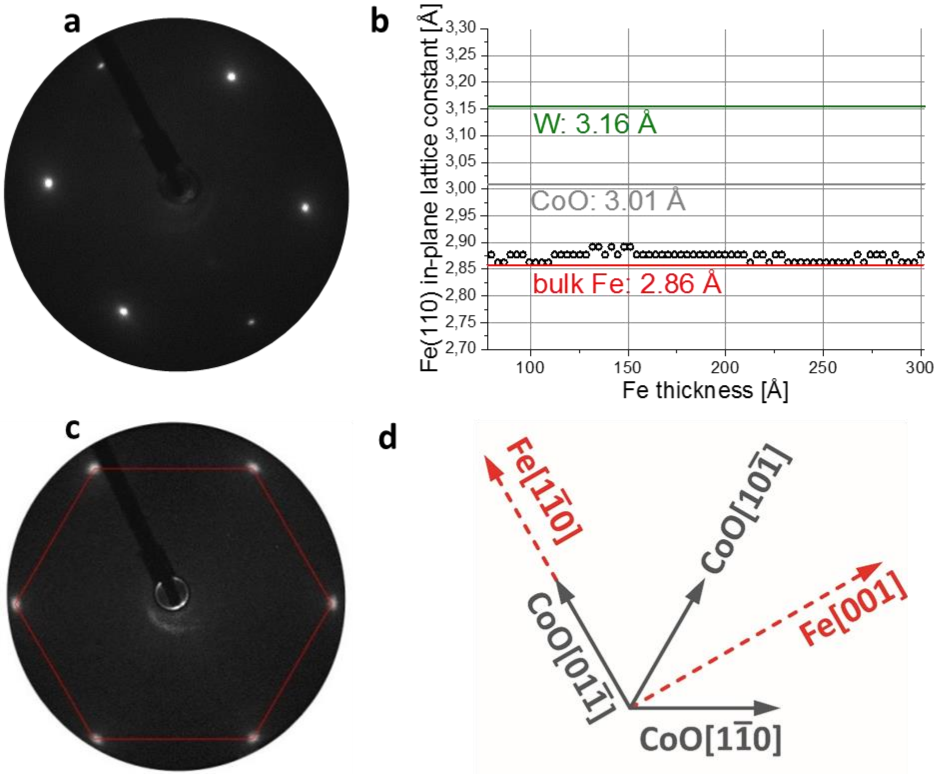

Fig. 1 **(a)** LEED pattern (electron energy E = 120 eV) from the Fe(110) surface. **(b)** Fe thickness dependence of the in-plane lattice constant of Fe(110) film as determined from LEED spot positions. **(c)** LEED pattern of a 90 Å layer of CoO deposited on Fe(110)/W(110), E = 40 eV. The regular hexagon marks the positions of the diffraction spots. **(d)** Schematic sketch showing the in-plane crystallographic directions of Fe(110) and CoO(111) surfaces, as concluded from LEED images.
